# Improving Pore Characteristics, Mechanical Properties and Thermal Performances of Near-Net Shape Manufacturing Phenolic Resin Aerogels

**DOI:** 10.3390/polym16111593

**Published:** 2024-06-04

**Authors:** Ruyi Sha, Jixiang Dai, Bingzhu Wang, Jianjun Sha

**Affiliations:** 1 Key Laboratory of Advanced Technology for Aerospace Vehicles of Liaoning Province, Dalian University of Technology, Dalian 116024, China; sharuyi@mail.dlut.edu.cn (R.S.);; 2 State Key Laboratory of Structural Analysis, Optimization and CAE Software for Industrial Equipment, Dalian University of Technology, Dalian 116024, China

**Keywords:** phenolic resin aerogel, porous material, ambient pressure drying, thermal insulation, fire retardancy

## Abstract

Thermally stable high-performance phenolic resin aerogels (PRAs) are of great interest for thermal insulation because of their light weight, fire retardancy and low thermal conductivity. However, the drawbacks of PRA synthesis, such as long processing time, inherent brittleness and significant shrinkage during drying, greatly restrict their wide applications. In this work, PRAs were synthesized at ambient pressure through a near-net shape manufacturing technique, where boron-containing thermosetting phenolic resin (BPR) was introduced into the conventional linear phenolic resin (LPR) to improve the pore characteristics, mechanical properties and thermal performances. Compared with the traditional LPR-synthesized aerogel, the processing time and the linear shrinkage rate during the drying of the PRAs could be significantly reduced, which was attributed to the enhanced rigidity and the unique bimodal pore size distribution. Furthermore, no catastrophic failure and almost no mechanical degradation were observed on the PRAs, even with a compressive strain of up to 60% at temperatures ranging from 25 to 200 °C, indicating low brittleness and excellent thermo-mechanical stability. The PRAs also showed outstanding fire retardancy. On the other hand, the PRAs with a density of 0.194 g/cm^3^ possessed a high Young’s modulus of 12.85 MPa and a low thermal conductivity of 0.038 W/(m·K).

## 1. Introduction

Due to their low density, high porosity and ultra-low thermal conductivity, aerogels have been considered as potential lightweight thermal insulation materials [1,2]. Particularly, phenolic resin aerogels (PRAs) have the merits of low cost, fire retardancy and excellent thermal insulation performance, and therefore, they have become attractive thermal insulation materials in civil industries [3,4]. Furthermore, the thermal isolation performance of PRAs through ablation also endows them with applications in the thermal shield of re-entry flight vehicles [5,6,7]. Particularly, for the application of high-temperature thermal protection systems, PRAs not only consume and emit aerodynamic heat but also block the conduction of heat, making them promising and effective lightweight ablative materials [8,9,10]. On the other hand, PRAs with low thermal conductivity also play a vital role in energy conservation by protecting thermal energy from excessive dissipation [11,12]. Despite the numerous advantages of PRAs, there are still some issues, such as low preparation efficiency, large volumetric shrinkage and poor mechanical performance, which restrict their wide applications.

Generally, the synthesis of PRAs starts from the preparation of the phenolic resin wet gels by polymerizing the phenol and formaldehyde [13] or the linear phenolic resin and hexamethylenetetramine [14]. Recently, the PRAs were also synthesized by using the linear boron phenolic resin as the raw materials, which exhibited better thermal stability than those of the aerogels synthesized by the LPR [15]. On the other hand, to obtain the PRAs, the solvent must always be removed from phenolic resin wet gels without destroying their original structural integrity. The traditional drying methods include supercritical drying (SCD) [16,17] and ambient pressure drying (APD) [18,19]. Although the SCD method can obtain the PRAs with suitable pore structure and performance, the synthesis process is complex, and the cost is high. On the contrary, the APD method is much simpler than the SCD, but the porous structure of PRAs is easily broken by the large capillary force generated by solvent evaporation during drying, leading to a high requirement for the robustness of wet gels. Furthermore, the significant volumetric shrinkage of wet gels during drying is also a critical issue, which is not conducive to near-net shape manufacturing and performance control.

To overcome the above-mentioned issues that occurred in the APD process, different reinforcements have been introduced to enhance the rigidity of phenolic resin wet gel, such as polymer fiber felts [20,21], carbon fiber felts [22,23] and ceramic fiber felts [24,25]. The introduction of these reinforcements makes the synthesis of PRAs easier and inhibits the shrinkage of wet gels. Specifically, the shrinkage rate of pure PRAs is as large as 24.8–32.7%, while it can be significantly reduced to 4.5–7.6% by introducing the reinforcement [25]. However, the reinforcement also leads to a significant increase in density and the thermal conductivity of aerogels. Specifically, the thermal conductivity of aerogel composites can be 1.44 times that of pure aerogels [26]. On the other hand, due to the mismatched modulus between the aerogels and the reinforcements, the reinforcements may also exacerbate the brittleness of PRAs, thus generating cracks in PRAs when suffering from large deformations. It was found that the composite PRAs presented structural damage at a compressive strain of less than 10% [6,27], while the pure PRAs can withstand the compressive strain of up to 25–50% [28]. Another way to inhibit shrinkage during drying is to increase the shrinkage resistance of phenolic resin wet gel itself by enhancing the rigidity and modulating the pore size distribution. Generally, the rigidity can be enhanced by increasing the polymerization time, temperature and pressure [29]. And the pore size distribution can be modulated by adjusting the raw material ratios [30]. However, without the reinforcements, shrinkage during drying is still a serious problem in the synthesis of lightweight PRAs [31,32].

In the current work, in order to decrease the shrinkage rate and improve the pore characteristics, mechanical properties and thermal performances of PRAs, a high-efficiency polymerization and near-net shape manufacturing technique was developed to prepare the PRAs through the synergistic effect of linear phenolic resin (LPR) and boron-containing thermosetting phenolic resin (BPR), where hexamethylenetetramine (HMTA) was used as the curing agent to crosslink the LPR and BPR. Then, the shrinkages, microstructures, pore characteristics, mechanical properties and thermal performances of PRAs were characterized. Based on the preliminary results, the influence mechanisms of the BPR on the comprehensive performances of PRAs were revealed.

## 2. Materials and Methods

### 2.1. Materials

Linear phenolic resin (LPR: supplied by Henan Hengyuan New Material Co., Ltd., Xinyang, China), boron-containing thermosetting phenolic resin (BPR: supplied by Anhui Tianyu High-temperature Resin Material Co., Ltd., Bengbu, China) and hexamethylenetetramine (HMTA: supplied by Tianjin Damao Chemical Reagent Co., Ltd., Tianjin, China) were used as raw materials. Herein, the BPR aimed to enhance the rigidity and modulate the pore size distribution of PRAs, and the HMTA was used as the curing agent to crosslink the LPR and the BPR. Additionally, ethylene glycol (EG) and anhydrous ethanol (EtOH) were used as solvents.

### 2.2. Synthesis of PRAs

**Figure 1:**
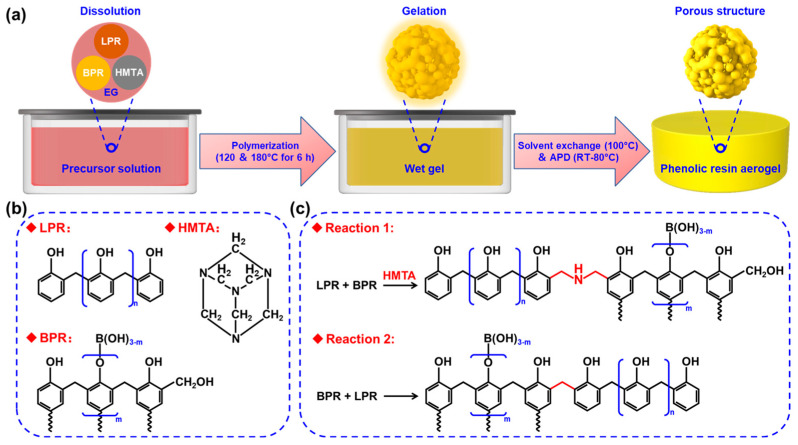
Synthesis of PRAs: (**a**) flow chart of the synthesis of PRAs; (**b**) molecular structures of LPR, BPR and HMTA; (**c**) chemical reactions of LPR, BPR and HMTA during polymerization.

A typical synthesis procedure of PRAs is presented in Figure 1a. Firstly, the phenolic resins (LPR and BPR) and HMTA with different mixing ratios were dissolved in EG solvent to prepare the precursor solution, followed by a high-efficiency polymerization process (120 °C for 3 h and subsequently 180 °C for another 3 h) to obtain the wet gels. Then, the EG solvent in the wet gels was exchanged with EtOH on a heated platform (100 °C). Finally, the PRAs with ultra-low shrinkage rates were obtained by drying the wet gels under ambient pressure (RT for 12 h, 40 °C for 6 h and subsequently 80 °C).

Figure 1b shows the molecular structures of LPR, BPR and HMTA. The corresponding chemical reactions during the high-efficiency polymerization process are shown in Figure 1c. Firstly, the C-N bonds in the HMTA were opened at high temperatures (≥120 °C) and then reacted with the highly active ortho- and para-hydrogen atoms of the phenolic hydroxyl groups, which resulted in the crosslink between the LPR and the BPR (reaction 1) [20]. Furthermore, the BPR belongs to the thermosetting phenolic resin, and the -CH_2_OH groups in the BPR could also react with the ortho- and para-hydrogen atoms of the phenolic hydroxyl groups through dehydration condensation, thus resulting in the crosslink between the LPR and the BPR (reaction 2) [29]. However, the -CH_2_OH groups might also react with themselves through dehydration condensation to form unstable ether linkages. Therefore, the polymerization temperature was then increased to 180 °C to convert the ether linkages into stable methylene [33]. Such chemical reactions together contributed to the high-efficiency polymerization of PRAs. As a result, the polymerization time in this work could be shortened to 6 h for synthesizing the high-performance PRAs.

### 2.3. Characterization

The density was calculated based on the weight and dimensions of the aerogels. In the current work, the cylinder specimens were used for the density measurement. The linear shrinkage rate was calculated based on the diameter of cylinder aerogels before and after the APD process. The microstructure was observed by a field-emission scanning electron microscope (NOVA NanoSEM 450, FEI, Hillsborough, OR, USA) with an acceleration voltage of 3 kV and a spot of 3–3.5. The pore characteristic was carried out by mercury intrusion porosimetry (AutoPore IV 9500, Micromeritics Instrument Corp., Atlanta, GA, USA) with a pressure range from 0.1 to 61,000 psia. The thermal conductivity was measured by a hot wire method (TC-3200, Xiatech, Xi’an, China) at room temperature according to the international standard ASTM D5930 [34]. The transmission performance of aerogels was evaluated by a KBr disk method on a Fourier transform infrared spectrometer (FTIR: Thermo Nicolet IS50, Thermo Fisher, Waltham, MA, USA), and the wavenumber ranged from 4000 to 400 cm^−1^. Thermogravimetric analysis (TGA/DSC3+, Mettler Toledo, Zurich, Switzerland) was performed to analyze the thermal stability of PRAs at temperatures ranging from 25 to 900 °C in a nitrogen atmosphere, and the heating rate was 10 °C/min. The compression test at 25, 100 and 200 °C was carried out by a universal mechanical testing machine (WDW-100, Changchun Fangrui, Changchun, China) with a compression rate of 5 mm/min, and the dimensions of the aerogels were 10 mm in diameter and 7 mm in thickness. The Young’s modulus was calculated based on the slope of the compressive stress–strain curves at the elastic stage, and at least five specimens were used for each test.

## 3. Results and Discussion

### 3.1. Shrinkage of Wet Gels

As shown in Table 1, PRAs with different mixing ratios of raw materials were synthesized. For comparison, the PRAs synthesized with pure LPR and pure BPR were also synthesized and used as the controlled specimens, which were denoted as LA and BA, respectively. Then, the LPR- and BPR-containing PRAs were synthesized by adjusting the HMTA concentrations and denoted as LBA1–LBA4. It should be noted that the total concentration of phenolic resin in the precursor solution was kept constant.

**Figure 2:**
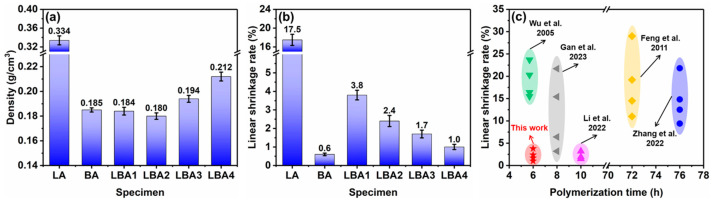
Density and linear shrinkage rate of PRAs: (**a**) density of PRAs; (**b**) linear shrinkage rate of PRAs; (**c**) comparison of linear shrinkage rate and polymerization time of different PRAs [4,29,31,32,35].

The density and the linear shrinkage rate of PRAs were measured as presented in Figure 2a,b. The density of LA was 0.334 g/cm^3^ (Figure 2a), which was much larger than other PRAs. This was due to the large shrinkage rate of LA, which was 17.5%, as shown in Figure 2b. The density of BA was 0.185 g/cm^3^, corresponding to the lowest linear shrinkage rate of 0.6%. As for the LBA1–LBA4, the linear shrinkage rates decreased with increasing HMTA concentration. The linear shrinkage rates were 3.8% for LBA1, 2.4% for LBA2, 1.7% for LBA3 and 1% for LBA4. In contrast, the densities of LBA1–LBA4 showed a different tendency, which reduced first and then increased with increasing HMTA concentration. The densities of LBA1–LBA4 were 0.184, 0.180, 0.194 and 0.212 g/cm^3^, respectively. This phenomenon indicated that increasing the concentration of HMTA could promote the polymerization reactions of PRAs (Figure 1c), thus causing the increasing weight and density.

Figure 2c shows the comparison of linear shrinkage rate and polymerization time of LBA-series PRAs with other openly reported PRAs. To reduce the shrinkage during drying, different strategies have been adopted, such as using the drying–curing method [4], drying by SCD method [31], using fiber as reinforcements [29,32], using the high-pressure-assisted polymerization method (2–8 MPa) [29] and exchanging solvent with low surface tension (n-heptane) [35]. In comparison, both the high-efficiency polymerization and low shrinkage were achieved on the LBA-series PRAs.

**Figure 3:**
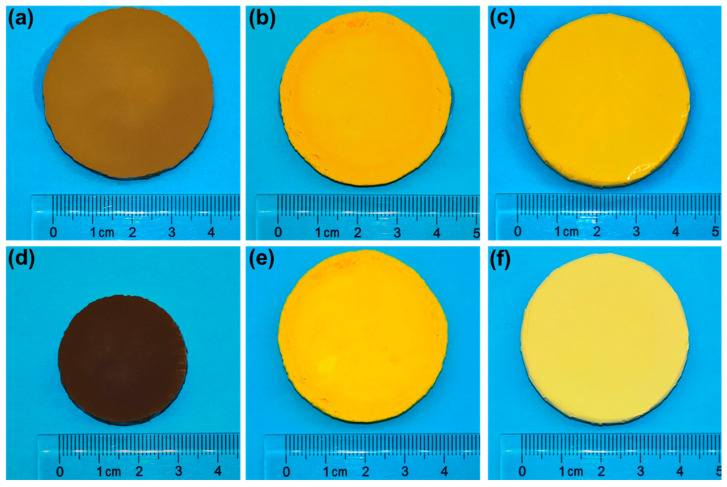
Optical images of PRAs: wet gels of (**a**) LA, (**b**) BA and (**c**) LBA3 before drying; aerogels of (**d**) LA, (**e**) BA and (**f**) LBA3 after drying.

Figure 3a–f show the optical images of LA, BA and LBA3 before and after drying at ambient pressure. The shrinkage of BA and LBA3 was almost invisible, while it was very significant for LA. The large shrinkage rate of LA indicated that the high-efficiency polymerization approach developed in this work was inapplicable to LA.

**Figure 4:**
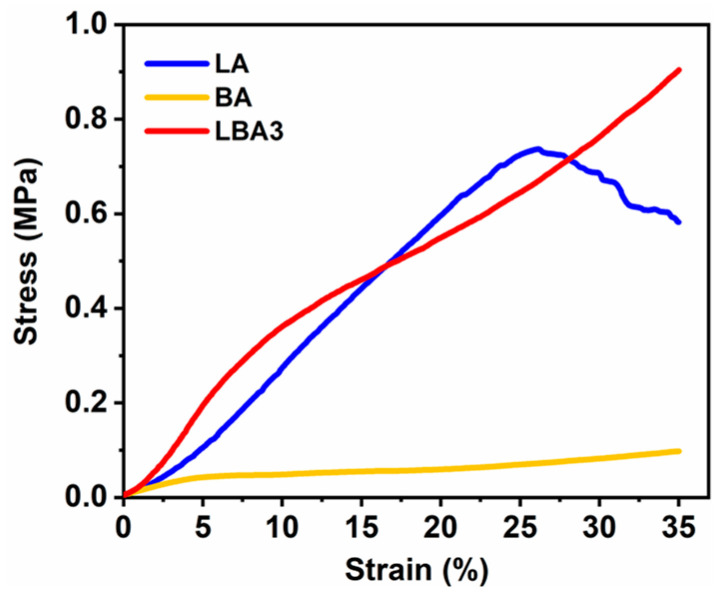
Compressive stress–strain curves of the wet gels of LA, BA and LBA3.

The shrinkage behavior of the wet gels during drying was similar to a compression process caused by their own weight and capillary force (generated by solvent evaporation). Therefore, the shrinkage resistance of wet gels might be associated with their rigidity. As a result, the wet gels with higher rigidity could have higher shrinkage resistance during drying. To find the reason for the different shrinkage resistance, the compression test was performed on the wet gels of LA, BA and LBA3, as shown in Figure 4. Different slopes of stress–strain curves at the initial linear part were observed, which corresponded to the elastic modulus of wet gels. It was clear that the LBA3 wet gel showed a much higher rigidity than those of the LA and BA wet gels, thus ensuring a lower shrinkage deformation during drying.

Furthermore, the LA wet gel showed a different compressive behavior compared with BA and LBA3 wet gel, where partial structure damage occurred at a strain of about 21.5%. However, the BA and LBA3 wet gel exhibited suitable flexibility. This might be associated with the addition of BPR [36,37]. On the other hand, the BA wet gel presented the lowest shrinkage rate, but its rigidity was much lower than that of the LBA3 wet gel, implying that the pore characteristic should also be responsible for the shrinkage of wet gels. Therefore, we further characterized the microstructure and pore characteristics to reveal the governing factors as follows.

### 3.2. Microstructure

**Figure 5:**
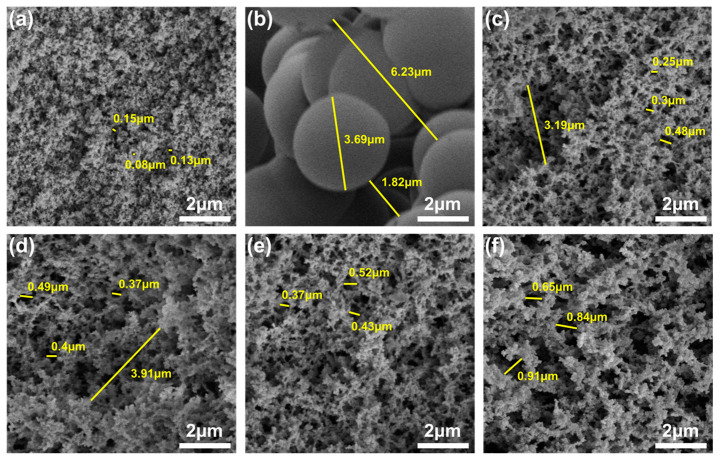
Microstructures of PRAs from SEM observation: (**a**) LA; (**b**) BA; (**c**) LBA1; (**d**) LBA2; (**e**) LBA3; (**f**) LBA4.

Figure 5 shows the microstructures of PRAs. The LA possessed a relatively dense and fine particle microstructure (Figure 5a). The particle diameter and pore size of LA were about 50 nm and 0.08–0.15 μm, respectively. However, the BA showed a quite different microstructure, where the beads-stacked porous structure was typical, and the particle diameter (~3.69 μm) and pore size (1.82–6.23 μm) were very large (Figure 5b). As for the LBA1–LBA4 series (Figure 5c–f), they were synthesized by a mixture of LPR and BPR; herein, the constant LPR/BPR ratio was used, but the HMTA concentration was varied. The particle diameters of LBA1–LBA4 were about 65, 75, 85 and 100 nm, respectively. However, the 3D network structures of LBA1 and LBA2 (HMTA concentration: 0.017 and 0.021 g/mL, respectively) were not well developed and not completely interconnected spatially, thus leading to the partial collapse of the 3D networks (Figure 5c,d). As a result, huge and deep holes with widths of about 3.19 and 3.91 μm were observed on the LBA1 and LBA2, respectively. Furthermore, the pore size of LBA1 and LBA2 were about 0.25–0.48 and 0.37–0.49 μm, respectively. In the case of LBA3 (HMTA concentration: 0.03 g/mL), it exhibited a homogeneous and nanoparticles-assembled fibrous 3D network structure (Figure 5e). And the pore size of LBA3 was about 0.37–0.52 μm. However, the coarsening of aerogel particles occurred in LBA4 (HMTA concentration: 0.05 g/mL), and big voids (pore size: 0.65–0.91 μm) were also found, as shown in Figure 5f.

The porous structure affected not only the shrinkage but also the mechanical performance and the thermal insulation performance of PRAs [38]. The large shrinkage rate of LA could be associated with the low rigidity and the small pore size. Particularly, the small pore size of LA caused a severe capillary force generated by the solvent evaporation during drying [39]. Therefore, the LA wet gel with a low rigidity could not withstand the severe capillary force, thus resulting in an extremely high shrinkage rate, substantially increased density and significantly reduced porosity. In contrast to LA, the BA was composed of large pores, which were conducive to exhausting the solvent. Furthermore, the capillary force generated by the solvent evaporation during drying could also be significantly reduced. As a result, the BA possessed an ultra-low shrinkage rate. By mixing the LPR with the BPR, the porous microstructure was regulated, and a very low shrinkage rate for LBA serious aerogels was also achieved through the synergistic effect of LPR and BPR, as shown in Figure 2b.

### 3.3. Pore Characteristic

**Figure 6:**
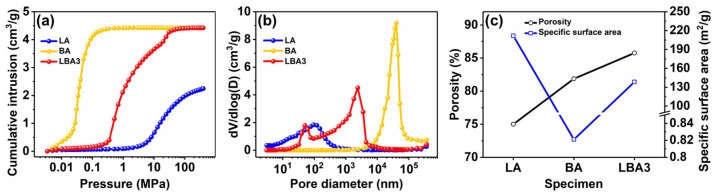
Pore characteristics of LA, BA and LBA3: (**a**) mercury intrusion curves; (**b**) pore size distribution; (**c**) porosity and specific surface area.

Apparently, the shrinkage of wet gels should be related to the pore characteristic, which was significantly affected by the type of phenolic resins. Therefore, the mercury intrusion method was applied to determine the influence of phenolic resin types on the pore characteristic, as shown in Figure 6.

Figure 6a shows the mercury intrusion curves. It was clear that the mercury could infiltrate into BA even if the pressure was low (<0.1 MPa), indicating the existence of large pores in the BA. In contrast to BA, the mercury began to infiltrate into LA at the high-pressure stage (>1 MPa). Due to the large shrinkage rate of LA, the cumulative mercury intrusion content was low. The pressure of mercury intrusion in LBA3 was located between LA and BA (0.1–1 MPa), while the cumulative mercury intrusion content of LBA3 was similar to BA.

Figure 6b shows the distribution of pore size in different PRAs. The peaks of pore size distribution were located at 91.5 nm for LA and 39.9 μm for BA, respectively. However, the LBA3 presented a unique pore characteristic, which showed a bimodal pore size distribution. The peaks of the pore size distribution of LBA3 were located at 50.4 nm and 2.4 μm, respectively. Therefore, the unique bimodal pore characteristic should contribute to the excellent shrinkage resistance of LBA3.

Figure 6c shows the porosity and specific surface area of different PRAs. The porosities of LA, BA and LBA3 were 75, 81.9 and 85.8%, respectively. The specific surface area of LA was up to 211.7 m^2^/g, which was much larger than those of the BA (0.82 m^2^/g) and the LBA3 (138.5 m^2^/g). The significantly different specific surface areas should be associated with their network structures. As mentioned above, the networks of LA were composed of fine particles, and the shape of the particles was irregular (Figure 5a). Therefore, the fine and irregular particles endowed the LA with a quite large specific surface area. In contrast to LA, the BA showed huge and regular spherical particles (Figure 5b). As a result, the specific surface area of BA was quite small. As for the LBA3, the particle size of LBA3 was between the LA and BA (Figure 5e), and therefore, the specific surface area of LBA3 was also between the LA and BA.

### 3.4. Mechanical Property

**Figure 7:**
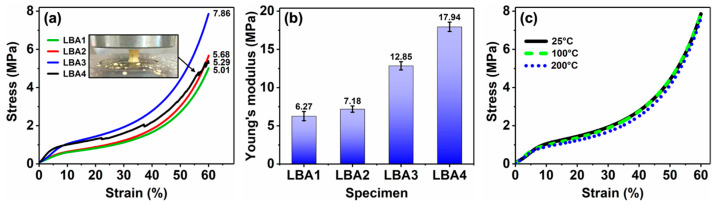
Mechanical properties of PRAs: (**a**) compressive stress–strain curves of LBA1–LBA4, and the image of LBA4 after the compression test (inset in **a**); (**b**) Young’s modulus of LBA1–LBA4; (**c**) compressive stress–strain curves of LBA3 at 25, 100 and 200 °C.

Figure 7a shows the compressive stress–strain curves of LBA1–LBA4 with increasing HMTA concentration. When the HMTA concentration was no more than 0.03 g/mL, the compressive stress of aerogels increased with increasing HMTA concentration. The compressive stresses at the 60% strain were 5.01 MPa for LBA1, 5.68 MPa for LBA2 and 7.86 MPa for LBA3, respectively, while it was 5.29 MPa for LBA4. Obviously, the LBA3 showed the highest compressive stress, which should be attributed to the robust and fibrous 3D network structures (Figure 5e). The compressive stress of LBA3 at 60% strain was about 1.38 times higher than those of LBA1, LBA2 and LBA4.

It should be noted that zigzags were observed in the stress–strain curve of LBA4 at the strain of 22.1%. As shown in the inset of Figure 7a, partial structure damage occurred for the LBA4 after the compression test. Such a phenomenon could be associated with the collapse of the porous microstructures in LBA4 composed of coarse particles and big voids (Figure 5f). During compression, the stress would concentrate around the voids, causing damage to the partial network structure and resulting in a sudden decrease in stress. As shown in Figure 7b, with the increase in HMTA concentration, the rigidity of LBA1–LBA4 was enhanced. The Young’s modulus was about 6.27 MPa for LBA1, 7.18 MPa for LBA2, 12.85 MPa for LBA3 and 17.94 MPa for LBA4, respectively. However, the high rigidity of LBA4 exacerbated its brittleness. A similar phenomenon could be observed in the literature [28].

According to the above-presented results, clearly, by mixing the LPR with the BPR and adjusting the HMTA concentration, improved mechanical properties have been achieved in PRAs, where the LBA3 stood out. Therefore, due to the excellent mechanical properties of LBA3, we further investigated the thermo-mechanical stability. Figure 7c shows compressive stress–strain curves of LBA3 at elevated temperatures in an ambient atmosphere. From 25 to 200 °C, the compressive behaviors of LBA3 were very similar. After the compression test at 200 °C, the geometric structure of LBA3 still kept integrity even at the compressive strain of 60%, indicating excellent thermo-mechanical stability.

### 3.5. Thermal Performance

**Figure 8:**
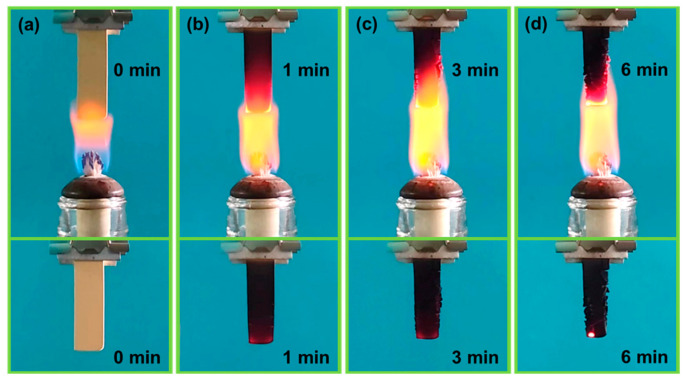
Optical images of LBA3 exposed to an alcohol flame: (**a**) 0 min; (**b**) 1 min; (**c**) 3 min; (**d**) 6 min.

Except for the mechanical properties, fire retardancy and thermal stability are also essential for PRAs as a thermal insulation material. Figure 8 shows the fire-retardant performance of LBA3, which was determined by putting it in an alcohol flame (~600 °C). With increasing the exposure time, the appearance of LBA3 gradually changed from the original yellow to black, and a carbonated protective layer was observed on the surface, as shown in Figure 8a–d. A similar phenomenon was also observed in the literature [40]. Furthermore, no softening, burning or heavy smog was observed at the different times when the specimen was moved away from the flame, indicating excellent fire retardancy.

**Figure 9:**
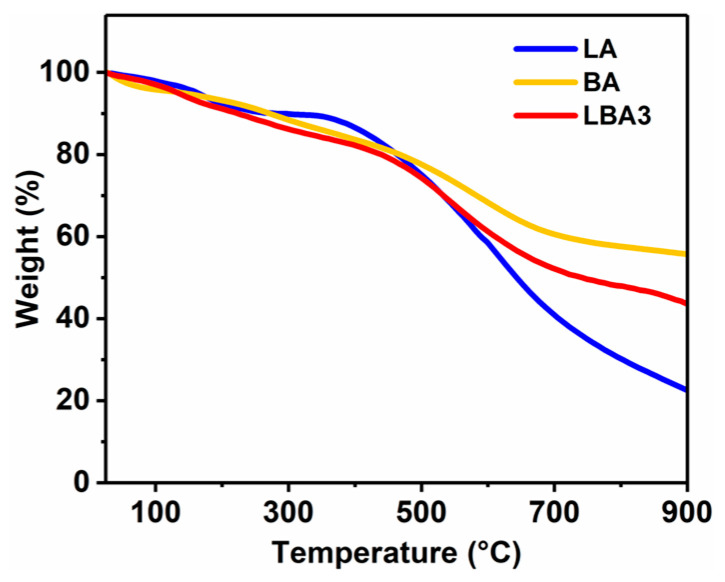
TGA curves of LA, BA and LBA3.

The thermal stability of LBA3 was determined from RT to 900 °C through a TGA test, as shown in Figure 9, where LA and BA were used as the control specimens. It was found that the weight of the three PRAs underwent a sharp decrease in the range of 300–700 °C due to the pyrolysis of phenolic resin. As the temperature reached 300 °C, the dehydration reactions of methylene and hydroxyl groups happened, and water was released [41]. Furthermore, hydrogen, carbon monoxide and carbon dioxide were also released by decarbonization, dehydrogenation and deoxidation reactions, which together led to a sharp decrease in weight [15]. After the TGA test, the weight loss was 77.4% for LA, 56.4% for LBA3 and 44.3% for BA, respectively. The weight loss followed the order: LA > LBA3 > BA, which showed an opposite change tendency with the BPR content in PRAs, proving that the BPR was beneficial to improving the thermal stability of PRAs.

**Figure 10:**
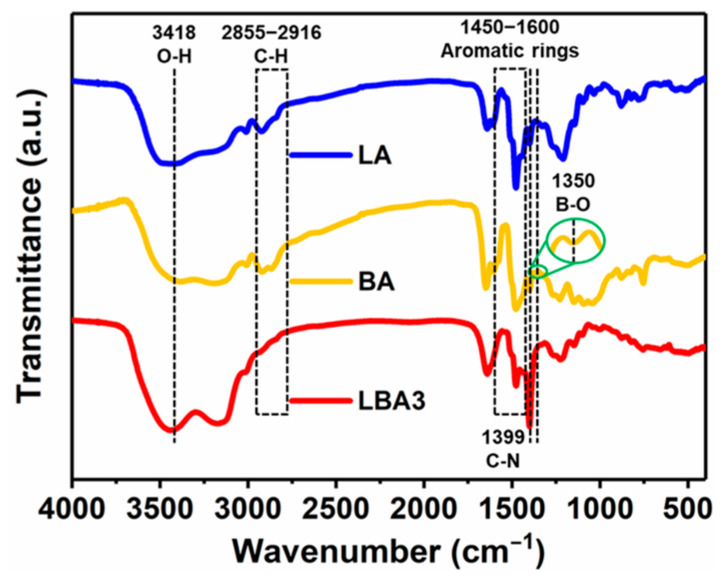
FTIR curves of LA, BA and LBA3.

The excellent fire retardancy and thermal stability of PRAs should be strongly associated with their chemical bonds. Therefore, to determine the influence mechanism of BPR on the thermal performances of PRAs, the FTIR spectrum was measured, as shown in Figure 10. The broad peak of 3418 cm^−1^ was associated with O-H stretching vibrations [42]. The absorption peaks between 2855 and 2916 cm^−1^ resulted from C-H stretching vibrations [43]. The peaks between 1450 and 1600 cm^−1^ were attributed to the stretching vibrations of aromatic rings [44]. And the absorption peak at 1399 cm^−1^ was corresponding to the vibrations of C-N [30]. Therefore, according to the chemical bonds in PRAs, it could be inferred that when PRAs were exposed to a flame, noncombustible gases such as water vapor, nitrogen oxides and carbon dioxide would be generated, which could take away the heat and reduce the oxygen content, thus endowing the PRAs with a high oxygen index. A similar phenomenon was also found in the literature [45].

On the other hand, the abundant carbon-containing chemical bonds also contributed to the high carbon content of PRAs, which could transform into the carbonated protective layer at high temperatures that isolated oxygen and heat (Figure 8) [46]. Furthermore, the B-O bonds at the 1350 cm^−1^ absorption peak (Figure 10) also possessed beneficial impacts on the formation of pyrolytic carbon [47]. It was found that the B-O bonds could stabilize the terminal benzene rings from scission at high temperatures, thus endowing the PRAs with excellent thermal stability (Figure 9). A similar phenomenon could be observed in the literature [48]. Additionally, the high residual pyrolytic carbon caused by the B-O bonds further enhanced the fire retardancy of PRAs. It also demonstrated that the B-O bonds could enhance the thermo-mechanical properties of PRAs (Figure 7c).

### 3.6. Thermal Conductivity

**Figure 11:**
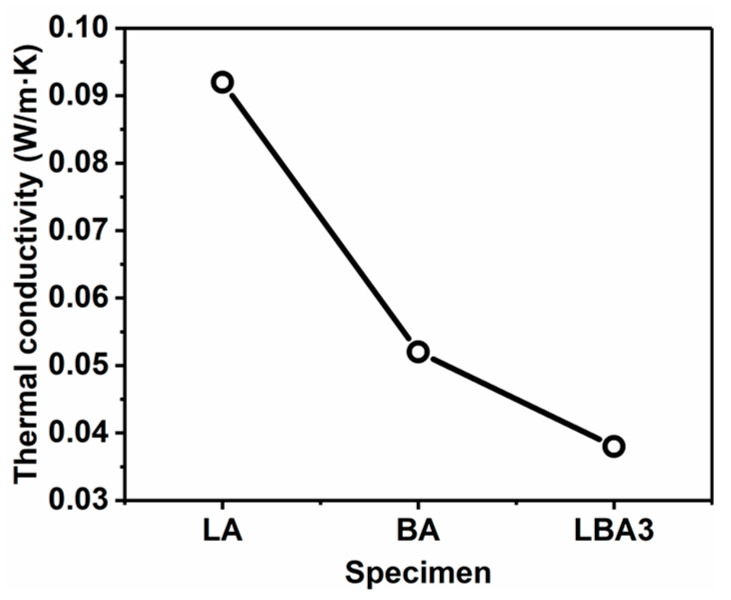
Thermal conductivity of LA, BA and LBA3.

Figure 11 shows the thermal conductivity of PRAs with different typical porous structures. The thermal conductivities of LA, BA and LBA3 were 0.092, 0.052 and 0.038 W/(m·K), respectively. There were three heat transfer paths in PRAs: thermal conduction along solid networks and gas, thermal convection by gas movement and thermal radiation [26]. The thermal radiation was negligible at room temperature [38]. Therefore, thermal conduction and convection should be responsible for the different thermal conductivities of PRAs. In comparison, the LA showed quite high density due to the large shrinkage rate, which resulted in abundant solid heat transfer paths per unit volume. As a result, the thermal conductivity of LA was much higher than the BA and the LBA3.

On the other hand, it was found that the density of BA was lower than the LBA3, while the thermal conductivity showed an opposite tendency. Two factors should be responsible for this phenomenon. Firstly, the huge particles in BA made the heat transfer along the solid networks much easier compared with the tortuous solid networks in LBA3. Secondly, the large pores in BA also significantly increased the convective heat transfer efficiency, thus resulting in high thermal conductivity [49]. Therefore, to pursue efficient thermal insulation performance, both the high porosity and nano-scale microstructure would be favorable to PRAs, which could result in the few solid heat transfer paths and the trapped air in the 3D network structures. As a result, the thermal conductivity of LBA3 was quite low.

## 4. Conclusions

Near-net shape manufacturing of PRAs was realized by a high-efficiency and low-shrinkage process. PRAs with improved pore characteristics, mechanical properties and thermal performances were achieved through the synergistic effect of LPR and BPR. The main conclusions are summarized as follows.

(1) Through the synergistic effect of LPR and BPR, the shrinkage rate of PRAs can be significantly reduced. The shrinkage rate is dependent on the ratio of raw materials, which varied from 17.5 to 1.7%. The low shrinkage rate is attributed to the enhanced rigidity and the unique bimodal pore size distribution. The bimodal pore size distribution (50.4 nm and 2.4 μm) can effectively decrease the capillary force generated by the solvent evaporation during drying.
(2) The HMTA can affect the porous structure of PRAs. When the HMTA concentration is 0.03 g/mL, the PRAs present a homogeneous and nanoparticles-assembled fibrous 3D network structure. Such a porous structure endows the PRAs with excellent thermal insulation performance. As a result, the PRAs with a density of 0.194 g/cm^3^ possess a low thermal conductivity of 0.038 W/(m·K).
(3) The PRAs possess outstanding thermo-mechanical stability and almost have no mechanical degradation from 25 to 200 °C. The PRAs can bear a stress of 7.86 MPa without failure at a compressive strain of 60% at 25 °C. Furthermore, the PRAs also show excellent fire retardancy and thermal stability. They are incombustible when exposed to a flame (~600 °C for 6 min). And the obvious pyrolysis starts at temperatures beyond 300 °C based on the TGA test.

## Figures and Tables

**Table 1:** The raw material concentration of PRAs.

Specimen	LPR (g/mL)	BPR (g/mL)	HMTA (g/mL)
LA	0.193	0	0.03
BA	0	0.193	0.03
LBA1	0.15	0.043	0.017
LBA2	0.15	0.043	0.021
LBA3	0.15	0.043	0.03
LBA4	0.15	0.043	0.05

## Data Availability

Data are contained within the article.
